# Fabrication of Human Keratinocyte Cell Clusters for Skin Graft Applications by Templating Water-in-Water Pickering Emulsions

**DOI:** 10.3390/biomimetics4030050

**Published:** 2019-07-11

**Authors:** Sevde B. G. Celik, Sébastien R. Dominici, Benjamin W. Filby, Anupam A. K. Das, Leigh A. Madden, Vesselin N. Paunov

**Affiliations:** 1 Department of Chemistry and Biochemistry, University of Hull, Hull HU6 7RX, UK; 2 Department of Biomedical Science, University of Hull, Hull HU6 7RX, UK

**Keywords:** tissue engineering, spheroids, keratinocyte, HaCaT, water-in-water emulsions, Pickering emulsions, PEO, DEX, hydrogels, alginate

## Abstract

Most current methods for the preparation of tissue spheroids require complex materials, involve tedious physical steps and are generally not scalable. We report a novel alternative, which is both inexpensive and up-scalable, to produce large quantities of viable human keratinocyte cell clusters (clusteroids). The method is based on a two-phase aqueous system of incompatible polymers forming a stable water-in-water (w/w) emulsion, which enabled us to rapidly fabricate cell clusteroids from HaCaT cells. We used w/w Pickering emulsion from aqueous solutions of the polymers dextran (DEX) and polyethylene oxide (PEO) and a particle stabilizer based on whey protein (WP). The HaCaT cells clearly preferred to distribute into the DEX-rich phase and this property was utilized to encapsulate them in the water-in-water (DEX-in-PEO) emulsion drops then osmotically shrank to compress them into clusters. Prepared formulations of HaCaT keratinocyte clusteroids in alginate hydrogel were grown where the cells percolated to mimic 3D tissue. The HaCaT cell clusteroids grew faster in the alginate film compared to the individual cells formulated in the same matrix. This methodology could potentially be utilised in biomedical applications.

## 1. Introduction

Tissue engineering (TE) for regenerative medicine represents a challenge for the biomedical field with a wide-range of applications, including repair, replacement and regeneration of damaged tissues and organs [1]. A lack of available organs for transplants or tissue reconstruction slows down recovery of multiple patients. Moreover, major risks of these procedures include infection or rejection [2]. TE usually needs three components: Cells, scaffolds and growth factors [3]. Three-dimensional (3D) cell culture allows better results as the microenvironment is represented more accurately [4]. Cells usually interact with each other in the extracellular matrix (ECM), a non-cellular scaffold, which plays an important role in multiple processes such as cell proliferation, differentiation or migration [5,6] To mimic these conditions, different methods of 3D cell culture were developed: (i) Scaffold based: Polymeric hard scaffolds, biological scaffolds, micro patterned surface microplates and (ii) non-scaffold based: Microfluidic 3D cell culture, hanging-drop microplates, spheroids microplates containing ultra-low attachment (ULA) coating [7] The two last techniques mentioned are used for the production of cell spheroids, along with suspension culture in bioreactor (spinner flask, microgravity bioreactor) and micro-patterned surfaces [8,9] Cell spheroids are based on the self-assembling properties of cells, which leads to 3D cell aggregates [10]. The low cost of these techniques, their ability to generate complex cellular structures and the accuracy of the cell microenvironment represent a promising method for 3D cell culture [8,11] despite challenges in controlling their size and avoiding core necrosis [10]. Fibroblast cell spheroids can be formed using cell clustering processes where they adhere to each other rather than to a substrate. This involves methods such as spinner culture, NASA rotary culture and non-adhesive surfaces [9]. Size control of the individual spheroids was reported by using the hanging-drop culture technique [12,13,14,15,16], 3D culturing in microwells [17], micro-rotational flow [18] and the magneto-Archimedes effect [19]. Recently reported methods for the production of cellosomes [20,21] can also serve as a possible route for the larger scale production of desired spheroids. Encapsulation of cells in such structures has also been done using natural biopolymers [22]. Most of these methods are very limited by the amount of cell spheroids that can be produced. Nguyen et al. [23] and Ganley et al. [24] showed that water-in-water Pickering emulsions could be stabilized by solid particles. A more comprehensive review on stabilization of w/w Pickering emulsion with colloid particles was published by Dickinson [25]. Singh et al. [26] also reported encapsulation of viable microbial cells probiotic bacteria in carboxymethyl cellulose-gelatin water-in-water emulsions.

Aqueous two-phase systems (ATPS) have been previously used to create cancer spheroids [27,28] primarily by dispensing dextran rich drops (DEX) with tumour cells into polyethylene glycol rich media (PEG). The dispersed tumour cells have been grown in these DEX drops to form individual spheroids, which were further studied for response to drug treatments [27,28,29,30] As may be expected a higher resistance to chemotherapy agents was observed when spheroids created with this technique were compared to 2D culture [30]. The techniques used previously produced effective tumour spheroids to study drug effects, however, the aim of those studies was to create large spheroids, which by their nature should have a necrotic core and proliferating cells on the spheroid surface. However, for tissue engineering (TE) applications, the need to dispense individual DEX drops with cells into the PEG phase practically limits the scalability of these methods in their scope to produce large quantities of cell spheroids [27,28,29,30]. Recently, Das et al. [31] reported a rapid and scalable production technique for tissue spheroids of human embryonic kidney cells (HEK 293) by trapping them in w/w emulsion droplets stabilized by protein particles. Their technique is based on w/w emulsion formation where the cells effectively distribute in the emulsion droplets, which are stabilised by protein particles. This method is based on using water-in-water droplets to encapsulate the cells, and then shrinking the droplets by changing the ATPS equilibrium, which rapidly drives the cells to form large clusters that are harvested by breaking down of the w/w emulsion.

In the current study, we aimed to form not true spheroids but dense clusters of cells (termed here as clusteroids) that could have downstream applications where preserving cell viability is required such as skin grafts. Human skin represents an important physical barrier protecting the internal environment from external attacks and regulating body homeostasis. Regenerative skin surgery requites a replacement of the epidermis, which consists mostly of keratinocytes, but also melanocytes and Langerhans cells [32,33]. TE uses cells distributed in naturally derived biocompatible hydrogels as scaffolds for 3D culturing due to their ability to retain water, provide structured environment suitable for cell growth and proliferation [34]. Various parameters are evaluated to provide the best conditions for cell culture, such as choice of polymer, concentration, molecular weight and chemical composition. Alginate is a polysaccharide composed of (1–4) β-d-mannuronic acid (M) units and α-l-glucuronic acid (G) units derived from brown algae and is used in the biomedical field for its biocompatibility, low toxicity, non-immunogenicity and low cost [35]. Crosslinking of sodium alginate hydrogels allows a mild gelation with cations such as Ca^2+^, which serve as ion junctions forming a 3D network by binding two COO^−^ groups. Calcium chloride is typically used as cross-linking agent, releasing Ca^2+^ ions in the hydrogels by a diffusion mechanism [36,37]. 

Here, we developed further the method of Das et al. [31] for the preparation of human keratinocyte cell clusters (clusteroids) using w/w Pickering emulsions as templates and explored their formulation in suitable hydrogel matrices for 3D tissue engineering of skin grafts. 3D cell cultures of human adult keratinocytes (HaCaT) cells were used for the production of spheroids [38]. HaCaT cells are known as non-tumorigenic and have been successfully transplanted on nude mice resulting in an epidermal tissue layer close to normal keratinocytes [39]. HaCaT cells can still express differentiation genes like keratin 1 and keratin 10, unlike keratinocyte cell lines virally transformed [39]. This makes HaCaT cell clusteroids an excellent model system for exploring their growth in alginate hydrogels as a 3D cell culture model.

**Figure 1:**
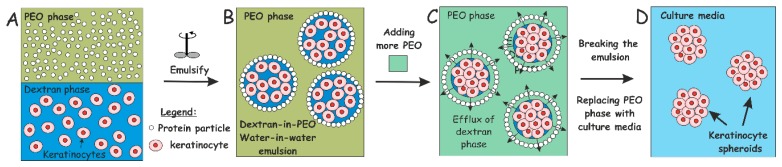
Schematics of our high-throughput method for preparation of keratinocyte cell spheroids (**A**–**D**). The keratinocyte cells are encapsulated in a dextran-PEO water-in-water emulsion template stabilised by 2 wt% WP particles. The continuous phase is PEO 5.5 wt% and the dispersed phase is composed of cells encapsulated in dextran droplets. Upon emulsification, cells prefer the discontinuous dextran phase, which allow their encapsulation. Adding more concentrated PEO phase causes osmotic shrinking of the cell-rich dextran drops, whose interfacial tension packs the cells into tissue spheroids. The latter are isolated by breaking the emulsion by dilution with culture media.

The schematics of our keratinocyte clusteroids fabrication method are briefly sketched in Figure 1. A water-in-water Pickering emulsion was formed to encapsulate the cells by mixing two aqueous solutions containing incompatible polymers: Polyethylene oxide (PEO) solution as a continuous phase and dextran solution (DEX) as a dispersed phase. The DEX-in-PEO emulsions were stabilized using whey protein (WP) particles. We found that the keratinocyte cells prefer to accumulate in the DEX phase, which allowed their effective encapsulation in the DEX emulsion drops. In this environment, they were further “compressed” against each other osmotically with the addition of more concentrated PEO phase to the emulsion and adhered to their neighbouring cells in the emulsion drops to form cell clusteroids. The formulation and growth in alginate hydrogel films as model skin grafts is schematically shown in the Supporting Information, Figure S1.

The paper is organized as follows. In the following section we discuss the materials and techniques used to control the size of the emulsion drop templates and the average size of the resulting keratinocyte clusteroids. The results section shows typical keratinocyte clusteroids produced by this technique and demonstrates that they consist of viable cells.

We also developed keratinocyte clusteroids composite formulation alginate gels and compare their growth with individual keratinocytes in the same scaffolding media. We found that HaCaT clusteroids can grow faster than the individual cells in alginate films. This methodology could be used as blueprint to enable growth of patients’ own skin cells and to fabricate autologous cell clusteroids for potential clinical applications.

## 2. Materials and Methods

### 2.1. Materials

Deionised water purified by reverse osmosis and ion exchange from a Milli-Q water system (Millipore) was used in all our studies. Its surface tension was 71.9 mN m^−1^ at 25 °C, with measured resistivity less than 18 mΩ cm^−1^. Dextran (MW 500 kDa) and PEO (MW 200 kDa) were both purchased from Sigma-Aldrich. Whey protein was sourced from (No1. Supplements, Suffolk, UK). Fluorescein diacetate (FDA, 98%) and sodium alginate and Corning^®^ Transwell^®^ polyester membrane cell culture inserts (12 mm, 12 well plates) were purchased from Sigma-Aldrich, UK. Sodium chloride (99.8%) and calcium chloride were purchased from Fisher Scientific. Dulbecco’s Modified Eagle Medium (DMEM) and Trypsin-EDTA were sourced from Gibco^®^, Fisher Scientific. NUNC Cell culture 24-well plates were purchased from Thermo Fisher Scientific.

### 2.2. 2D HaCaT Cell Culture 

HaCaT cell line culture was kindly provided by the Skin Research Group at St James University Hospital at Leeds and was cultured from samples frozen in liquid nitrogen. The cells were cultured in high-glucose DMEM media supplemented with 10% foetal bovine serum (FBS, Labtech, Heathfield, UK) and 1% antibiotics (Penicillin Streptomycin, Lonza) and placed in an incubator (37 °C, 5% CO_2_). After reaching 80% confluence, HaCaT cells were carefully washed with phosphate buffer saline (PBS) for 10 s then incubated with 0.25% Trypsin-EDTA (1X, Lonza), which allowed us to detach the cells from their support after 5 min. Its action was neutralized by adding complete DMEM medium before a centrifugation at 400× *g* for 4 min. After resuspension in fresh medium, the HaCaT cells were reseeded in tissue culture flasks (Sarstedt). All cell waste was left for at least 30 min in 1% Virkon viricidal disinfectant (Fisher Scientific) before its disposal.

### 2.3. Preparation of the Whey Protein (WP) Particles

Whey protein powder was dissolved in water at a concentration of 2 wt% for 2 h under agitation. The solution was placed at 4 °C for 12 h to hydrate the whey protein. Then, the solution was centrifuged at 10,800× *g* for 1 h and the supernatant was collected. A solution of 300 mM NaCl was prepared and mixed with an equal volume of WP solution. The pH was adjusted to 5.8 by drop-wise addition of filtered 0.5 M HCl aqueous solution. After heating the WP/NaCl solution in an oil bath at 85 °C for 15 min, it was left to cool at 4 °C. This precipitation process produced WP particles, which were used in our protocol as stabilizers for the water-in-water emulsions; these emulsions were used as a template for the encapsulation of the keratinocyte cells into clusteroids.

### 2.4. Production of w/w Pickering Emulsions, Cell Encapsulation and Clusteroid Isolation

PEO aqueous solution (5.5 wt%) was prepared by dissolving PEO into the heat-treated solution of WP, which constituted the continuous phase of the water-in-water emulsion. A centrifugation of the PEO solution was done beforehand at 5000× *g* for 7 min to remove the silica nanoparticles from the PEO solution, which were added by the manufacturer. A solution of 5.5 wt% dextran (in total) in high-glucose DMEM complete medium under sterile conditions was used as a disperse phase (DEX phase) together with the keratinocytes. The DEX phase with the keratinocytes formed typically a total volume fraction ф = 0.0909 with respect to the DEX/PEO w/w emulsion. To form the latter, the DEX phase (plus the cells) were transferred to the WP/NaCl/PEO solution and gently homogenized using two pumps with a BD Microlance™ 3 needle (21G ½, internal diameter 0.512 mm) and a BD Plastipak™ syringe of 5 mL by two pumps (Becton Dickinson, Wokingham, UK). The emulsion was prepared using PEO phase of volume fraction ф = 0.9181 and DEX phase (with the cells) of volume fraction (ф = 0.0909). This resulted in the cells encapsulation in the emulsion drops due to a higher affinity of the HaCaT cells to the DEX phase compared to the PEO phase. A more concentrated PEO solution was added to the emulsion (to adjust the final PEO concentration to a total of 8 wt%), which allowed the DEX droplets to shrink osmotically and obtain densely packed encapsulated HaCaT cells. The cell–cell adhesion in these structures resulted in the formation of cell clusteroids in approximately 15 min. To break the water-in-water emulsion and release the produced clusteroids, the solution was further diluted with high-glucose DMEM medium by a factor of 10. The clusteroids were left to sediment down by gravity for 15 min. After discarding the supernatant, clusteroids were carefully re-suspended in a fresh DMEM medium. 

### 2.5. Cell Viability Assay

Cell clusteroids were treated with a 5 g L^−1^ solution of fluorescein diacetate in acetone (10 μL per 1 mL of the dispersion of re-suspended clusteroids; Sigma-Aldrich) to evaluate cell viability. After 10 min of incubation at room temperature in the dark, the sample was observed under an Olympus BX-51 fluorescence microscope (Olympus) with a DP70 digital camera and FITC fluorescence filter set. Fluorescein diacetate (FDA) is known to diffuse through cell membranes, only viable cells are able to hydrolyse non-fluorescent FDA internally to fluorescein by intracellular esterase activity. Since fluorescein dissociates in water, it’s crossing of the cell membranes is hindered by its charge, which results in the accumulation of green fluorescent fluorescein inside intact cells [25,31,40]. This indicates that the cell membranes are intact, and the cells are viable.

### 2.6. 3D Keratinocyte Clusteroids Culture 

A solution of 1.5 wt% sodium alginate was prepared by dissolution in water and then sterilized in an autoclave. DMEM medium was mixed with the sodium alginate 1.5 wt% solutions at different volumes to vary the gelling agent concentration. HaCaT cell clusteroids were then carefully resuspended in this solution and seeded on 24-well tissue culture plates (Sarstedt). Addition of 2 wt% CaCl_2_ (aq) allowed crosslinking of the alginate chains in the media and formation of the hydrogel. After incubation, the CaCl_2_ solution was carefully pipetted out without compromising the integrity of the hydrogel-clusteroids composite and the wells were topped up with DMEM medium. 

### 2.7. Fabrication of HaCaT Cell Clusteroids

A sample of 0.3602 g wet weight (approx. 5.5 × 10^6^ cells) of the HaCaT cells was produced by centrifugation at 400× *g* for 4 min from the culture medium and re-suspended the cells in 540 μL of a solution of DMEM + Dextran 5.5 wt% (DEX dispersed phase). The cell volume fraction in the DEX phase was 0.25, i.e., approximately a quarter of the DEX emulsion drops volume was occupied by cells. Then, we added 1.295 mL of a WP solution to 2%/NaCl 300 mM + PEO 5.5 wt% (PEO continuous phase). The PEO concentration in the emulsion continuous phase was further increased (by adding 1.765 mL 10 wt% PEO in DMEM media) to shrink the DEX droplets with the cells and obtain HaCaT cell clusteroids. After 30 min, the emulsion was broken by 10-fold dilution with DMEM media to release the obtained clusteroids from the DEX drops. This procedure was used to isolate the HaCaT clusteroids prior to their imaging and viability characterisation in DMEM media.

### 2.8. Preparation of Model HaCaT Clusteroids in Alginate Hydrogels Formulations

A stock solution of 1.5 wt% sodium alginate was prepared by dissolution in water and then sterilized in an autoclave. A 0.5 wt% sodium alginate solution was prepared by mixing DMEM medium with the stock solution of 1.5 wt% sodium alginate solution. A 24-well plate was used to seed HaCaT clusteroids in sodium alginate hydrogels. HaCaT cells were resuspended in a solution 5.5 wt% Dextran in DMEM media (dispersed phase). 5.5 wt% PEO was added as a continuous phase and the emulsion was homogenized with a BD Microlance 3 needle (21G ½) and a BD Plastipak 5 mL syringe (needle internal diameter 0.512 mm) by two pumps. A more concentrated solution of PEO was used to shrink the cells in order to obtain good quality clusteroids. The sample was left to sediment over two hours after the emulsion was broken down by dilution with DMEM media. In the final formulation, the concentrations of sodium alginate and HaCaT cell clusteroids were 0.5 wt% and 0.12 g/mL, respectively. The composite alginate-HaCaT clusteroids formulations were seeded in the wells with a total volume of 100 µL. To crosslink the hydrogel matrix around the clusteroids, 100 µL of 2 wt% CaCl_2_ was added with a pipette on top of the alginate layer and was incubated for two hours in order to fortify the alginate hydrogel, after which the CaCl_2_ solution was carefully discarded without damaging the hydrogel. 1.00 mL of 90 vol% high-glucose DMEM medium supplemented with 10 vol% FBS and antibiotics was added in each well plate and the culturing was done at 37 °C over the course of seven days. The growth media was changed every two days. The growth of the clusteroids in the alginate film was monitored every day by taking multiple images by optical microscope in each well and the average size calculated after sizing a minimum 500 clusteroids from each well both in x-and y-direction.

### 2.9. SEM Imaging of HaCaT Clusteroids 

Scanning electron microscope (SEM) images of the clusteroids were taken in order to reveal their morphology. Samples were prepared for SEM images after fixing the cell clusteroids to avoid them to break apart during the evaporation of the aqueous solution. Of the HaCaT cell clusteroids in media, 1 mL was deposited on dry Aclar™ sheets (Agar Scientific Ltd., Essex, UK) or poly-lysine coated glass coverslips and treated with 2 wt% glutaraldehyde for 2 h. This was followed by washing with cacodylate buffer, rinsing with serial ethanol-water solutions of increasing ethanol concentration, starting from 50% ethanol and moving up to washing with an absolute ethanol and then drying by using a critical point dryer. In the case of clusteroids within an alginate film the 5 mm × 5 mm sample was washed with deionised water and deposited on the SEM stub and freeze dried at critical point temperature. The samples were imaged using a scanning electron microscope SEM (ZEISS EVO 60 EP-SEM).

### 2.10. Statistical Analysis 

Comparisons were made using unpaired *t*-tests or analysis of variance as appropriate with significance set at *p* ≤ 0.05. 

## 3. Results

### 3.1. WP Particle Characterisation

**Figure 2:**
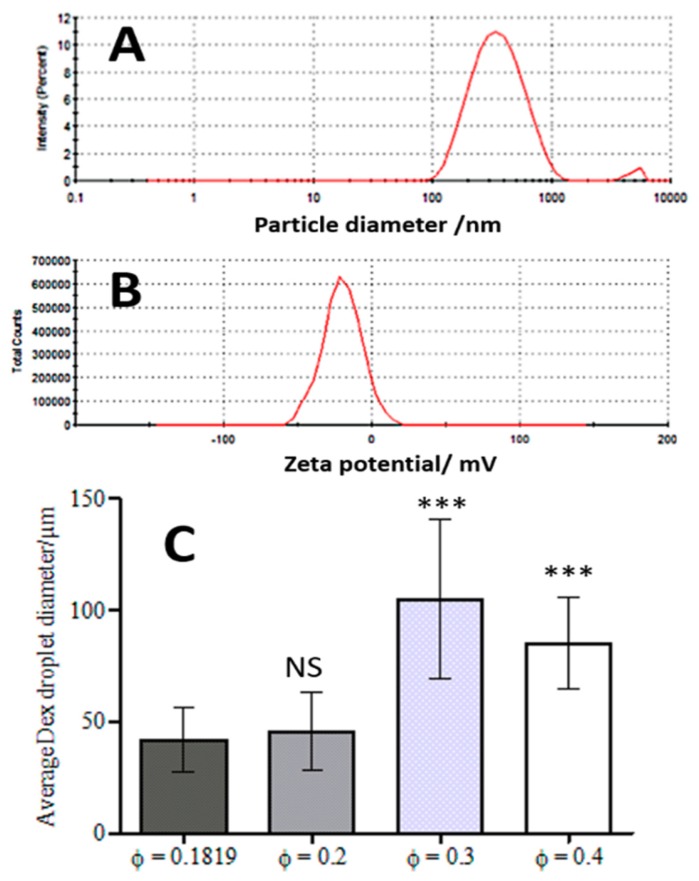
The average hydrodynamic diameter (**A**) and zeta-potential (**B**) for the produced WP particles at pH 5.8. (**C**) Average DEX droplet diameter for the DEX/PEO Pickering emulsion produced from WP/NaCl 300 mM solution at pH 5.8, 5.5 wt% PEO/5.5 wt% dextran for varying volume fractions of the Dextran phase (average of 200–300 individual drops). The data were obtained by optical microscopy measurements of the DEX droplets for each micrograph with Image J software. (Student’s *t*-Test, NS: Non-significant, *** *p* < 0.001).

Previous studies showed that emulsions made from preheated and aggregated proteins were more stable than the ones with native proteins [23,24]. The heat-treatment allows them to make aggregates that will adsorb at the interface between continuous and dispersed phase. Indeed, protein particles with a radius higher than 85 nm tended to increase droplet size [23]. WP particles produced by our methodology were characterised before using them to stabilise the w/w emulsions. Figure 2A shows typical size distribution of the WP particles where two peaks are observed. The main peak contains 98% of measured clusters with an average size of 305 ± 30 nm. The second, smaller peak shows a fraction of WP particles that were probably larger protein aggregates but were present at a much lower concentration. The emulsions were stabilized with a particle diameter in the region of 300 nm. Zeta potential gives information about the colloid stability of the nanoparticles. The WP particles measured zeta-potential of −20 ± 5 mV indicates a reasonably good colloid stability for the WP particles, which were used as stabilizers of the DEX/PEO Pickering emulsions (Figure 2B).

### 3.2. HaCaT Cell Encapsulation in w/w Emulsions

HaCaT cells (typically 50 vol% as a wet pellet) and 5.5 wt% Dextran solution (50 vol%) were mixed at the same volume fraction (ф = 0.0909 for the DEX phase and ф = 0.0909 for the cells) and the dispersed into the 5.5 wt% PEO phase by using two pumps with a syringe as described earlier. The volume fraction of the PEO phase was 0.8181. As a result, only few clusteroids were produced and many single cells were observed in the dispersion, which were not encapsulated in the DEX phase. Measurements with ImageJ Software (NIH, Bethesda, MA, USA) determined that the average droplet size was 37 ± 12 µm (Figure 2C). This did not change significantly upon increasing the concentration of the PEO phase, yielding small number of clusteroids of average size of 35 ± 8 µm. These results were not significantly different (Student’s *t*-Test, *p* < 0.05) and DEX droplets did not shrink enough to give compacted clusteroids. Besides, the size of the produced DEX droplets was too small to encapsulate the available amount of HaCaT cells.

### 3.3. Effect of the DEX Phase Volume Fraction

**Figure 3:**
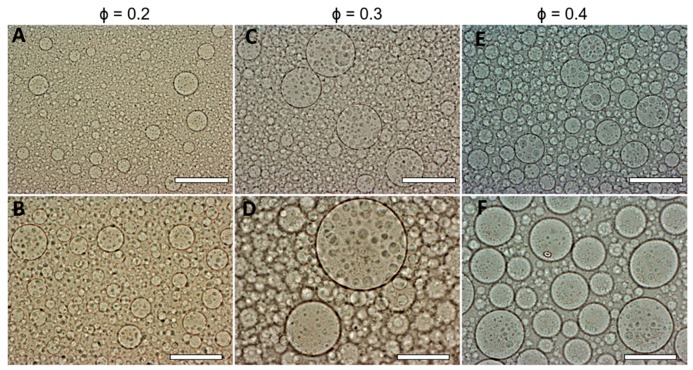
Optical microscopy images of a DEX/PEO water-in-water Pickering emulsion (PEO 5.5 wt% and DEX 5.5 wt%). (**A**,**B**) ф_Dex_ = 0.2, (**C**,**D**) ф_Dex_ = 0.3, (**E**,**F**) ф_DEX_ = 0.4 stabilized by 2 wt% WP particles at pH 5.8. Scale bars are (**A**,**C**,**E**) 200 µm and (**B**,**D**,**F**) 100 µm.

In order to increase the size of the DEX droplets, emulsions of different volume fractions of the DEX phase were produced: ф = 0.2, ф = 0.3 and ф = 0.4. The results shown in Figure 3 demonstrate the variation of the DEX phase volume has an impact on the emulsion droplet size. The average size of the DEX emulsion droplets with ф_DEX_ = 0.1819 and ф_PEO_ = 0.8181 was about 42 ± 14 µm. The volume fraction of the DEX phase that yielded the largest average drop size of 105 ± 36 µm was ф_DEX_ = 0.3 (Figure 2C). Hence, increasing the volume fraction of the DEX phase to 0.3 resulted in larger DEX droplets with the increased capacity for encapsulating a larger amount of HaCaT cells. At ф = 0.4 the observed droplets were decreased in size presumably due to instability and clusteroids did not consistently form. McClements and Jafari [41] demonstrated that the homogenization pressure had an impact on droplet size.

### 3.4. Effect of the w/w Pickering Emulsion Homogenization

The method used to homogenize the w/w emulsion had a direct influence on the final DEX phase droplet size. The use of a syringe allows a gentler homogenization and better control of the shear stress, which allowed us to control both the size and to certain extent the shape and morphology of the resulting clusteroids. This fine control was needed due to the ultra-low interfacial tension between the DEX phase and the PEO phase, which is easily overcome by the high shear and then it yields much smaller DEX droplet than the HaCaT cells. Results showed the size of the DEX droplets in the presence of the HaCaT cells also decreased with the number pumps with the syringe. These were done with syringe needle of internal diameter of 0.512 mm. However, there was no significant difference between the results of two or three pumps, where the DEX drop diameter levelled off at about 60 µm. However, a single pump was not enough to homogenize the DEX/PEO emulsion as the cells formed larger and more irregular clumps within the DEX droplets (see Supporting Information, Figure S2). The difference in the average droplet diameter between one and three pumps was statistically significant. However, microscopy examination revealed less cell clumping with two pumps than one. To keep droplets and clusteroids integrity, homogenization with the syringe was limited to two pumps for further experiments.

### 3.5. Effect of HaCaT Cell Volume Fraction and PEO Concentration

**Figure 4:**
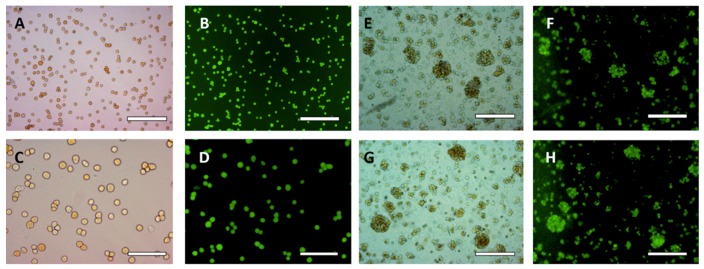
(**A**,**C**) Optical bright field images and (**B**,**D**) fluorescence microscope images of single HaCaT cells after being treated with FDA live/dead assay. (**E**,**G**) Optical bright field images and (**F**,**H**) fluorescence microscope images (**F**,**H**) of HaCaT cell clusteroids treated with FDA. FDA treatment was done on after the cell clusteroids fabrication. The fluorescence indicates that both the HaCaT cells preserve their viability during the clusteroids fabrication process, as described in Figure 1. Scale bars are (**A**,**B**,**E**,**F**,**G**,**H**) 200 μm and (**C**,**D**) 100 μm.

The HaCaT cell volume fraction in the DEX phase was varied from 0.2 (See Supporting Information, Figure S4) to 0.25 (See Supporting Information, Figure S5) in order to explore the effect of the larger concentration of cells in the DEX drops on their packing in cell clusteroids. The PEO final concentration used to shrink the DEX phase droplets with the encapsulated keratinocytes was increased to 10 wt% to allow a better compactness of the cell clusters into clusteroids. Clusteroids were produced with both cell volume fractions (Figure S4) and they shrank to more regular (nearly spherical) shapes with the increase of the PEO concentration from 5.5 wt% to 10 wt% for ф_HaCaT_ = 0.2 (See Supporting Information, Figure S6B). These results are significantly different from the control (ф_HaCaT_ = 0.0909, with transition from 5.5 wt% PEO to 8 wt% PEO), where the average DEX phase drop diameter decreased from 44 ± 13 µm to 39 ± 10 µm. For ф_HaCaT_ = 0.25, the reduction in the DEX drop diameter (with the cells) was not statistically significant (Figure S6C). The HaCaT cell viability was then estimated with an FDA live/dead assay [40,42]. Figure 4 demonstrates that the viability of the cells in the HaCaT clusteroids is not affected by an augmentation of PEO concentration. These results combined with the previous figure shows that ф_HaCaT_ = 0.2 and 10 wt% PEO as a concentration suitable for preparation and further growth of HaCaT cell clusteroids. This composition was used for further study.

### 3.6. Effect of the Volume Fractions of DEX Phase and HaCaT Cells

**Figure 5:**
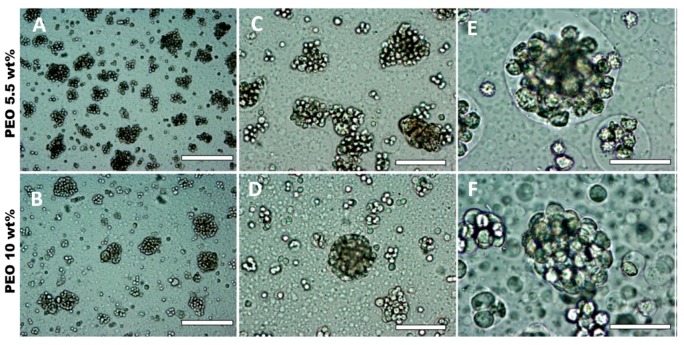
Optical microscopy images of (**A**,**C**,**E**) HaCaT cell droplets (5.5 wt% PEO/5.5 wt% Dextran) and (**B**,**D**,**F**) HaCaT cell clusteroids (10 wt% PEO/5.5 wt% Dextran) stabilized by 2 wt% WP particles. Here the cell and DEX volume fraction were, ф_HaCaT_ = 0.15 and ф_DEX_ = 0.25, respectively. Scale bars are (**A**,**B**) 200 µm, (**C**,**D**) 100 µm and (**E**,**F**) 50 µm.

**Figure 6:**
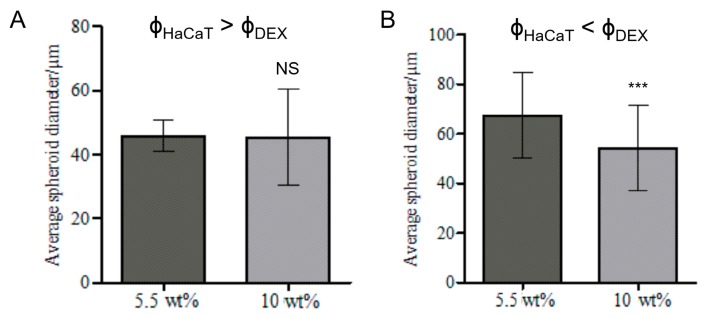
Average HaCaT spheroid diameter for emulsions 5.5 wt% PEO/5.5 wt% DEX or 10 wt% PEO/5.5 wt% DEX (**A**) ф_HaCaT_ = 0.25, ф_DEX_ = 0.2 and(**B**) ф_HaCaT_ = 0.15, ф_DEX_ = 0.25. The data were obtained by optical microscopy measurements of clusteroids for each micrograph with ImageJ Software (Student’s *t*-Test, NS: Non-significant, *** *p* < 0.001).

**Figure 7:**
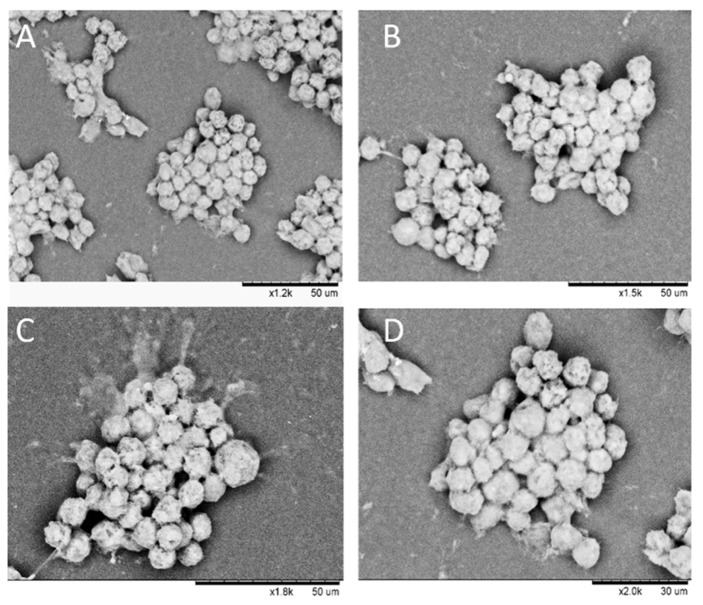
SEM images of a sample of HaCaT cell clusteroids after being removed from the medium, deposited on a glass substrate and freeze dried before imaging. (**A**–**D**) Images correspond to different resolutions. Note that the size of the clusters of cells is slightly lower than the original cell clusteroids due to shrinkage.

Various experiments were conducted to control the shape and size of HaCaT clusteroids. One was the addition of trypsin to temporarily stop the cells from sticking to each other too fast at different stages of the emulsion preparation. However, the results were inconclusive and HaCaT clusteroids tended to disintegrate in single cells after breaking the emulsion (data not shown) at equal initial volume fractions of HaCaT cells and DEX phase. In Figure S6 (Supporting Information), the HaCaT volume fraction was higher than DEX phase volume fraction (ф_HaCaT_ = 0.25, ф_Dex_ = 0.2). This resulted in cell clusteroids with more regular (round) shape but did not make a difference on the DEX drop diameters before and after the addition of more concentrated PEO phase (Figure 6A). On the contrary, for higher DEX volume fractions than the HaCaT cells volume fraction, a reduction of the average diameter from 67 ± 17 µm to 54 ± 17 µm was observed and this in turn yielded compacted cell clusteroids with a round shape (Figure 5 and Figure 6B). This difference is probably due to the overall Dextran phase concentration. By increasing the DEX phase volume fraction compared to that of HaCaT cells, the DEX droplets were larger and similar to the results shown in Figure 2 with ф_DEX_ = 0.3. This excess of DEX phase (Figure 5C) allowed more space for the cell rearrangement upon shrinking of the DEX droplets via osmotic pressure difference between these solutions. Clusteroids were then harvested by breaking the DEX/PEO emulsion by diluting with a DMEM medium by a factor 1 to 10 and allowed to sediment before resuspension of the clusteroids in fresh DMEM medium. Figure 7 shows typical SEM images of the isolated keratinocyte clusteroids and shows clustering of the adherent cells, although their size has been effectively reduced due to drying in the sample preparation procedure.

### 3.7. Hydrogel-HaCaT Spheroid Formulations 

#### 3.7.1. Effect of the Cell Spheroid Density 

HaCaT clusteroids were seeded on tissue culture plates at different cell densities. At low clusteroids cell concentration localization within the hydrogel could impede the formation of integrated tissue due to cells being too far from each other for percolation to occur. On the contrary, at too high initial cell concentration it is difficult to observe and monitor growth. Following the protocol for harvesting the clusteroids after breaking down the w/w emulsion, we found that upon mixing them with the alginate solution, most of the HaCaT clusteroids were disintegrated into single cells. Only a few of them remained stable but this was not enough to grow a tissue layer.

#### 3.7.2. Effect of the Breaking of the w/w Emulsion 

**Figure 8:**
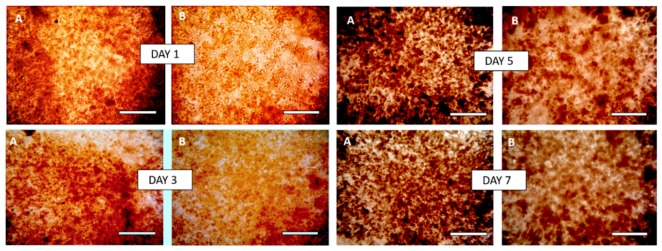
Bright field optical microscope images of HaCaT clusteroids isolated by a dilution of the DEX/PEO emulsion by a factor 3 with DMEM medium and incorporated with 0.75 wt% sodium alginate in DMEM media followed by cross-linking with 1M CaCl_2_. The HaCaT cells clusteroids were cultured in the alginate film for seven days under DMEM media and images were taken from each well to determine the average clusteroids size. Scale bars are 200 μm (**A**) and 100 μm (**B**) images. Clusteroids average size evolution is summarized in Figure 9.

**Figure 9:**
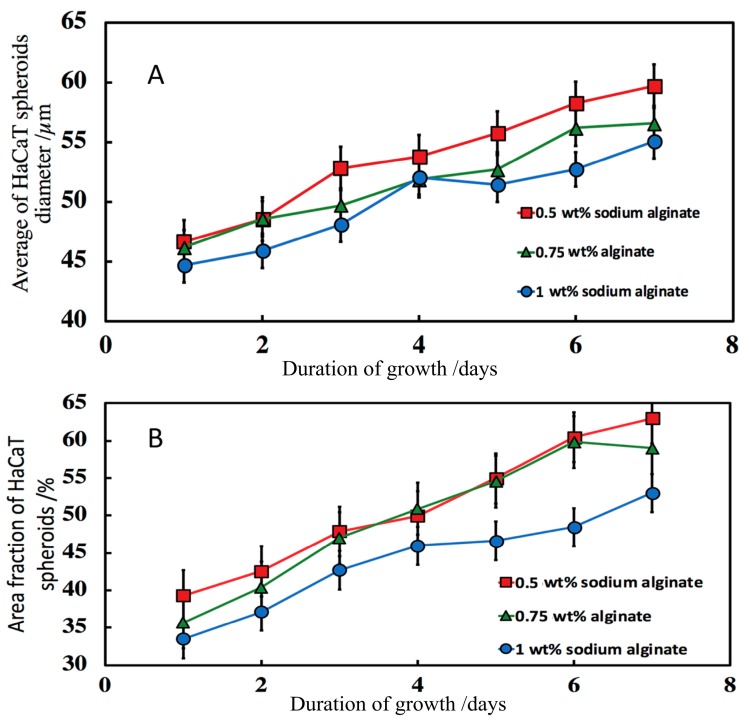
Evolution of the HaCaT clusteroids embedded in a hydrogel composed by different concentrations of sodium alginate and DMEM media for seven days. (**A**) The average spheroid size vs. time and (**B**) fractional area of the clusteroids in the alginate film vs. time. Measurements of the clusteroids were made every day with ImageJ software by taking the average of the vertical and horizontal diameter of 500 clusteroids in each well. The area fraction at 0.5 wt% and 0.75 wt% were both significantly different from the area fraction at 1% (*p* < 0.05).

To harvest HaCaT clusteroids and formulate them within the alginate solution without disintegrating them, a low shear method was designed. The DEX/PEO emulsion was broken by diluting it by a factor 3 in a solution of 0.75 wt% sodium alginate in DMEM media. Aliquots of the clusteroids suspension were dispensed in 12-well tissue culture plates and left to sediment for 3 h in the incubator at 25 °C before the supernatant being removed replaced by a fresh solution of 0.75 wt% sodium alginate in DMEM media. Compared to results obtained with the previous method, the HaCaT clusteroids were more stable and formed a uniform film in the alginate/DMEM media solution in wells, which was further fortified by addition of sterilized 1M CaCl_2_ solution over 30 min. The CaCl_2_ solution was carefully removed and replaced with fresh media. Measurements of the HaCaT cell clusteroids growth (as imaged in Figure 8) were taken to determine if there is or not an evolution of the tissue through a period of seven days. We measured the average HaCaT clusteroids size in samples from three separate wells, sizing a minimum of 500 clusteroids per well per day. Variations of the average spheroid size were observed, with a tendency to increase (Figure 9A). Image analysis was used to track the area occupied by the clusteroids in the alginate film throughout the cell growth, which also showed an upwards trend (Figure 9B), indicating that the cell clusteroids are expanding and after seven days come close to forming an integral tissue. We also completed a series of experiments of growing individual HaCaT cells integrated within alginate hydrogels at identical conditions as the ones with HaCaT cell clusteroids. The initial cell viability of the individual HaCaT cells was identical to this used to formulate the HaCaT clusteroids in the previous experiments. Figure S7 (Supporting Information) shows images of the alginate film with individual HaCaT cells over the course of several days. A small fraction of the original individual cells started developing and grow into new clusters of cells that are similar to the clusteroids. Figure S8 (Supporting Information) compares the average size of the keratinocyte clusteroids growing in 0.75 wt% alginate gels over four days with the size of spontaneously occurring spheroid cell clusters, formed in alginate films containing individual HaCaT cells. Note that these individual cells are suspended in the alginate gel rather than attached to the bottom of the well. The individual cells lost viability after four days of growth and the spontaneously occurring clusteroids grew at much lower rate than the pre-formed clusteroids by our method (see Figure S9). These results indicate that HaCaT cells grow well only when successfully attached to a solid substrate or another scaffolding material, as well as by attaching to their neighbouring cells in the clusteroids.

#### 3.7.3. Effect of the Sodium Alginate Concentration and the Calcium Chloride Solution Incubation Time

After less than a week of culture on well-plates, the 0.5 wt% alginate hydrogels-clusteroids composite film was found to spontaneously detach from the solid support. Therefore, the sodium alginate concentration was varied from 0.5 wt% to 1 wt% in order find the optimal conditions to strengthen the hydrogel matrix. At 1 wt% alginate and the CaCl_2_ solution incubation time of 10 min the micrographs did not show a significant growth between day 1 and day 3. After three days, the hydrogel-clusteroids film was no longer adhering to the bottom of the well-plate. In order to eliminate the possibility of poor cross-linking, we increased the incubation time of the hydrogel with the CaCl_2_ solution to 2 h. This approach seemingly resolved the film detachment problem [43].

After three days of growth, it looks like clusteroids percolated throughout the hydrogel to make contacts with cells from neighbouring clusteroids. We were unable to further monitor the clusteroids diameter beyond the seven days due to the percolation of the keratinocyte clusteroids and the formation of tissue (see Figure 8). Our working hypothesis was that if the hydrogel matrix was too strong and rigid, it would restrict the clusteroids growth and would slow down their percolation. In order to increase the growth rate, we formulated alginate-clusteroids films of lower concentration of alginate but prolonged incubation in CaCl_2_ solution (2 h) to allow more time for the Ca^2+^ ions to diffuse through the hydrogel matrix and strengthen the integrity of the hydrogel matrix. This allowed all grafts to remain attached to the well-plate during the growth process for more than seven days. Further experiments with trivalent cations cross-liking the keratinocyte clusteroids in alginate gels could be interesting but could also limit spheroid growth. Indeed, results from our experiments showed that hydrogels concentrated with 1 wt% sodium alginate delay HaCaT spheroid growth (Figure 9A).

### 3.8. Morphology of Composite Alginate Films with HaCaT Clusteroids

**Figure 10:**
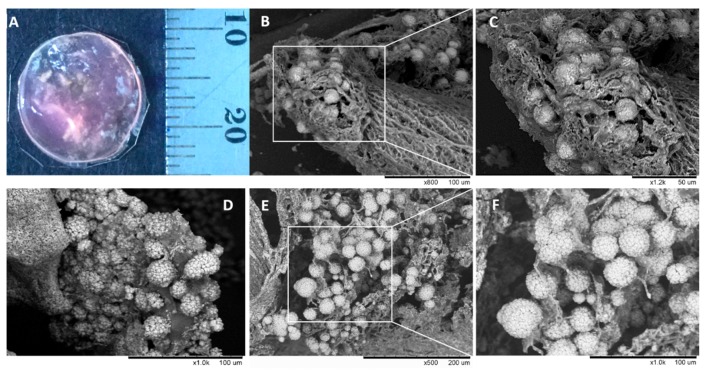
(**A**) Digital photograph of the HaCaT clusteroids within an alginate hydrogel film over four days. The film is collected from the bottom of the well plate. (**B**–**F**) SEM images of a freeze-dried sample of the film prepared from alginate gel-cultured HaCaT spheroid composite cultured in DMEM media for four days. (**C**,**F**) represent a zoom in of the SEM images (**B**,**E**), respectively.

The morphology of the prepared composite alginate films with HaCaT clusteroids is shown in Figure 10A. The digital photo is of the film as it is removed from the well and placed on solid support after been grown for four days. The SEM images of samples of such grafts after freeze drying at a critical point are shown at different resolutions in Figure 10B–F. One can see the clustering of the clusteroids in the hydrogel matrix and the way they have started to spread out and percolate. This process led to the formation of integral tissue over the course of seven days.

## 4. Discussion

We developed a new technique for the high throughput preparation of keratinocyte clusteroids with the aim of producing 3D tissues. HaCaT cells, a spontaneous immortalized human keratinocyte cell line, was used with the intention of growing skin, as keratinocyte are a major component of the epidermis [32]. This method is based on cell encapsulation within w/w Pickering emulsion droplets of an aqueous solution of Dextran in aqueous PEO solution stabilized by WP particles [31].

Our results showed that gentle homogenization with a syringe by two pumps was enough to avoid cell clumps and limit spheroid disintegration by shear stress. Increasing the HaCaT volume fraction to 0.2 and PEO final concentration to 10 wt% resulted in formation of more compact clusteroids, as shrinking of DEX droplets by osmotic pressure was more efficient. These droplets were about 2.5 times larger than the control with ф_DEX_ = 0.1819. This difference was statistically significant, as it was for the results with ф_DEX_ = 0.4. However, the average DEX droplet size was lower, 85 ± 20.8 µm. A DEX phase volume fraction of ф_DEX_ = 0.3 was selected for the fabrication of large HaCaT clusteroids by templating DEX/PEO Pickering emulsions.

After production of alginate films from keratinocyte clusteroids, various experiments could be elaborated to further control their proliferation and potential use as autologous skin grafts. A major issue with large spheroids is hypoxia, due to a decreased diffusion of oxygen and nutrients resulting in a necrotic core [4]. Hypoxia inducible factor (HIF)-1a, a survival factor among others, is likely to be activated in response to this lack of oxygen. Activation of these factors could be investigated in further experiments [44]. Studies have previously demonstrated that 3D skin models derived from HaCaT cells were lacking a stratum corneum and some abnormalities in protein expressions were detected like filaggrin, loricrin and involucrin [38]. Our method for fabrication of keratinocyte clusteroids could be adapted to primary cells directly derived from patients to produce autologous skin grafts.

The air–liquid interface (ALI) is a technique used to develop artificial skin. It consists of exposing keratinocytes to an ALI in order to induce their differentiation [45]. The quality of the artificial epidermis could be evaluated by detecting the expression of various specific keratins such as K5 and K14 for basal keratinocytes or K1, K10 and involucrin for late differentiation markers [46]. In case of deep wounds, a dermal substitute is used as a scaffold before applying and epidermal cover [32]. Bio-inks based on viable keratinocyte clusteroids could also potentially allow them to be bio-printed into customized 3D skin grafts where they can proliferate and grow more efficiently than single cells [47].

The in vitro culture of keratinocytes and fibroblasts for use in autologous grafts can take up to 21 days, during which time the patient must be cared for [48] although there was no relationship between admission and time to resection of tissue/application of grafts with hospital length of stay. The majority of graft procedures were carried out over 14 days after admission thus allowing enough time for in vitro cultured cells to potentially be available. It has also been noted that patients treated with neonatal fibroblasts for wound infection had a higher patient satisfaction and other parameters such as infection and graft take rates were comparable with allograft [49].

## 5. Conclusions

In this study we developed a rapid formation method to culture keratinocyte clusteroids by encapsulation of HaCaT cells in a water-in-water Pickering emulsion composed of polyethylene oxide (PEO) as the continuous phase and dextran (DEX) as the dispersed phase. Clusteroids were produced after increasing PEO phase concentration to shrink the dextran droplets via osmotic pressure. Various parameters were investigated, such as the volume fractions of dextran and the cells, emulsion homogenization and the final concentration of PEO. The method allows control of keratinocyte clusteroids formation from 40 μm to 200 μm depending on the shear applied to the emulsion and the DEX volume fraction. HaCaT clusteroids of 54 ± 17 μm were produced with volume fractions of: ф_DEX_ = 0.25, ф_HaCaT_ = 0.2 and ф_PEO_ = 0.6. The DEX/PEO w/w emulsion was homogenized by two pumps with a syringe and the PEO phase final concentration was brought to 10 wt%. After breaking the emulsion by diluting it by a factor of three with alginate solution in DMEM medium, isolated clusteroids were seeded in sodium alginate hydrogels at various concentrations fortified with Ca^2+^ ions. The clusteroids were grown in these hydrogels and allowed to percolate through and form an integral tissue, which may find applications within the skin graft field. 

## Supplementary Materials

The following are available online at https://www.mdpi.com/2313-7673/4/3/50/s1, Figure S1. Schematics of our high-throughput method for preparation of keratinocyte cell spheroids (A–D). Figure S2. Optical microscopy images of HaCaT cell droplets (PEO 5.5 wt%/DEX 5.5 wt%) stabilized by 2 wt% WP particles, at different stage of homogenization. Figure S3. Average DEX droplet diameter for the formulation of WP/NaCl 300mM solution at pH 5.8, PEO 5.5 wt%/DEX 5.5 wt% homogenized by 1, 2 or 3 pumps. Figure S4. Optical microscopy images of (A to C) HaCaT cell (ф = 0.2) droplets (PEO 5.5 wt%/DEX 5.5 wt%) and (D to F) HaCaT cell spheroids (PEO 10 wt%/DEX 5.5 wt%) stabilized by 2 wt% WP particles. Figure S5. Optical microscopy images of (A to C) HaCaT cell (ф = 0.25) droplets (PEO 5.5 wt%/DEX 5.5 wt%) and (D to F) HaCaT cell spheroids (PEO 10 wt%/DEX 5.5 wt%) stabilized by 2 wt% WP particles. Figure S6. Average HaCaT spheroid diameter for emulsions (A) PEO 5.5 wt or PEO 8wt% ф_HaCaT_ = 0.0909, (B) PEO 5.5 wt% or PEO 10 wt% ф_HaCaT_ = 0.2 and (C) PEO 5.5 wt% or PEO 10wt% ф_HaCaT_ = 0.25. Figure S7. Optical microscopy images of (A to C) HaCaT cell droplets (PEO 5.5 wt%/DEX 5.5 wt%) and (D to F) HaCaT cell spheroids (PEO 10 wt%/DEX 5.5 wt%) stabilized by 2 wt% WP particles. ф_HaCaT_ = 0.25 and ф_Dex_ = 0.2. Figure S8. Growth of individual HaCaT cells incorporated with 0.75 wt% sodium alginate in DMEM media followed by cross-linking with 1 M CaCl_2_. Figure S9. Growth curves of spheroids spontaneously formed from individual HaCaT cells (triangle symbols) and pre-formed HaCaT spheroids by our method (circle symbols) both incorporated within a film of 0.75 wt% sodium alginate in DMEM media followed by cross-linking with 1 M CaCl_2_.
