# Supplementary material for: Fabrication of Human Keratinocyte Cell Clusters for Skin Graft Applications by Templating Water-in-Water Pickering Emulsions
Source: Biomimetics (Basel). 2019 Jul 11;4(3):50. doi: 10.3390/biomimetics4030050 (PMC6784416; doi:10.3390/biomimetics4030050)
Supplement: Supplementary file 1 [file biomimetics-04-00050-s001.pdf]

Supplementary Information for:

Fabrication of human keratinocyte cell clusters for skin graft applications by templating water-in-water Pickering emulsions

Sevde B.G. Celik, [a] Sebastien R. Dominici,[a] Benjamin W. Filby,[a] Anupam A.K. Das,[a] Leigh A. Madden[b] and Vesselin N. Paunov[a]*

[a] *Department of Chemistry and Biochemistry,*

[b] *Department of Biomedical Science University of Hull, Hull, HU67RX, UK.*

**Corresponding author: Email: V.N.Paunov@hull.ac.uk*

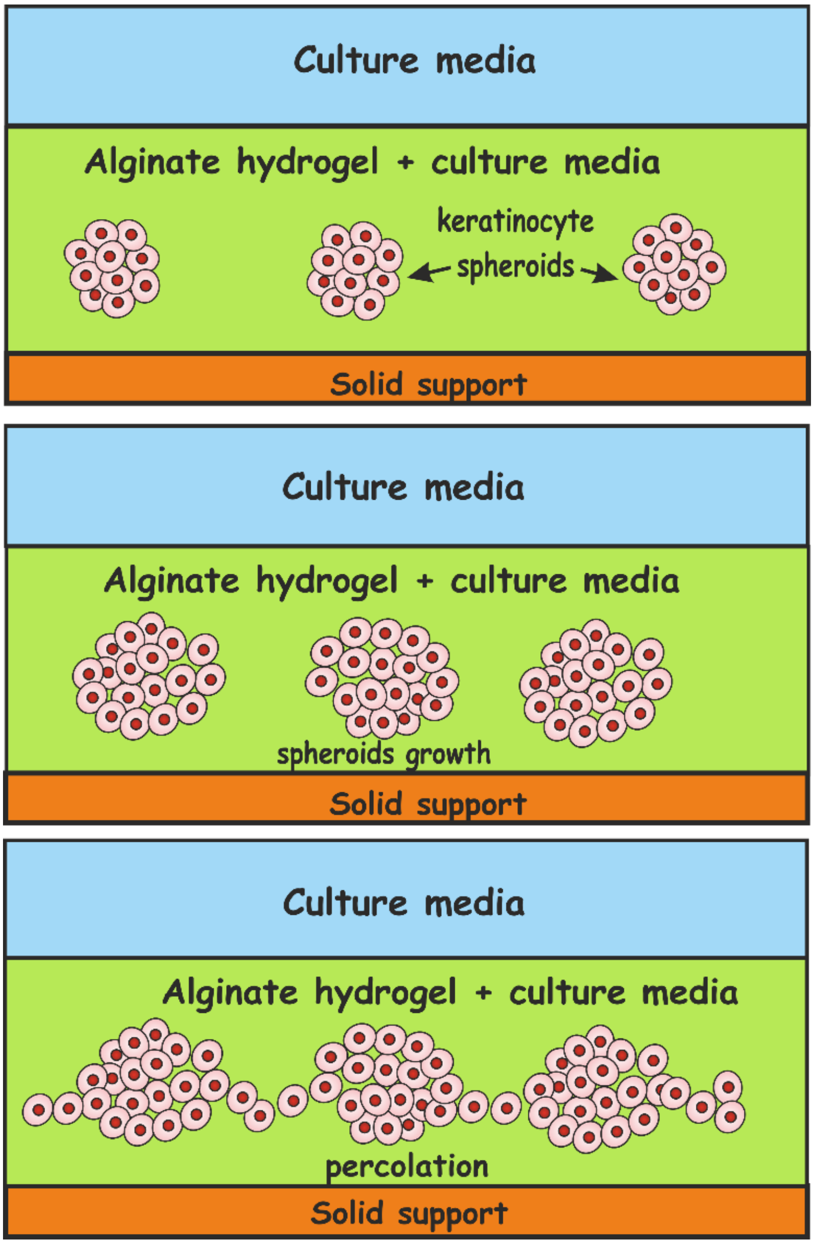

Figure S1. Schematics of the formulation of keratinocyte clusteroids in cross-linked alginate films and the evolution of their growth.

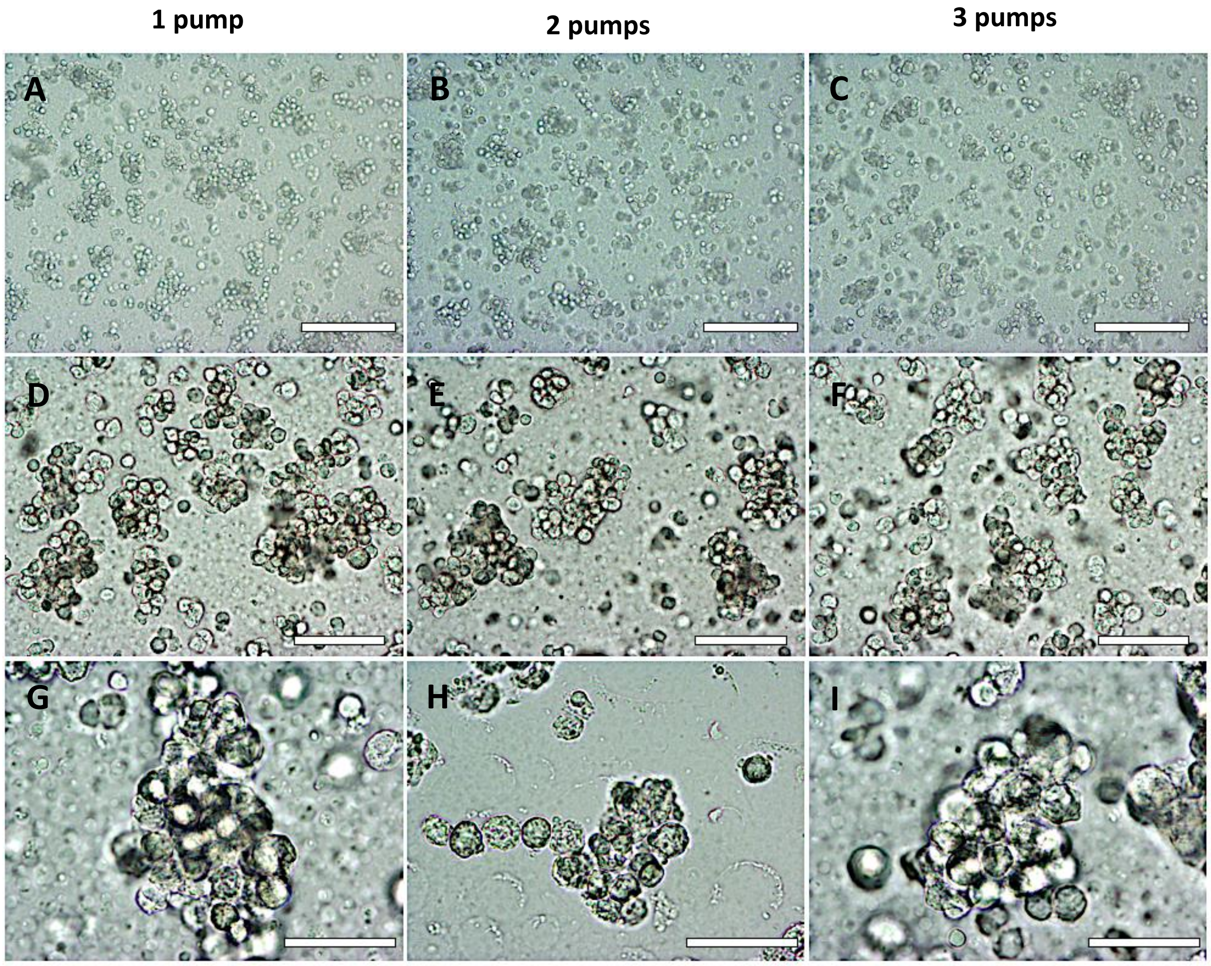

Figure S2. Optical microscopy images of HaCaT cell droplets (PEO 5.5 wt%/DEX 5.5 wt%) stabilized by 2wt% WP particles, at different stage of homogenization: (A, D, G) 1 pump, (B, E, H) 2 pumps and (C, F, I) 3 pumps. Scale bars are (A to C) 200 µm, (D to F) 100 µm and (G to I) 50 µm.

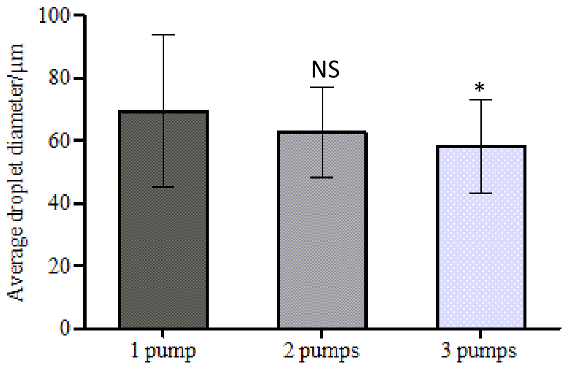

Figure S3. Average DEX droplet diameter for the formulation of WP/NaCl 300mM solution at pH 5.8, PEO 5.5 wt%/DEX 5.5 wt% homogenized by 1, 2 or 3 pumps. The data were obtained by optical microscopy measurements of the emulsion droplets for each micrograph with ImageJ Software. (Student's t-Test, NS: non-significant, *: $P < 0.05$)

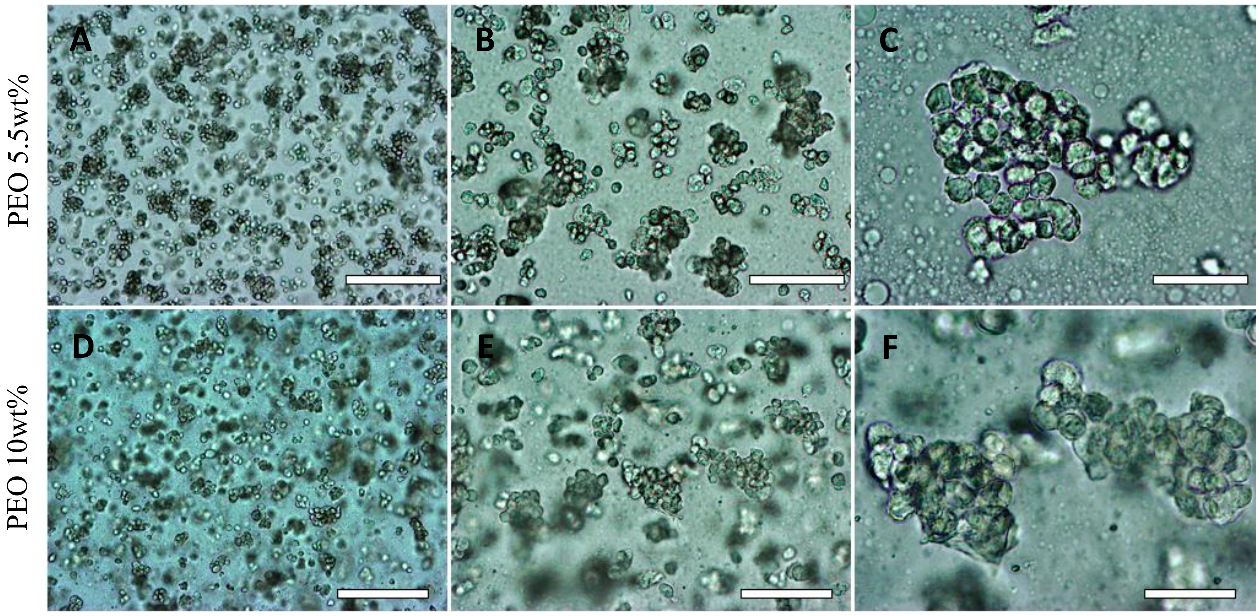

Figure S4. Optical microscopy images of (A to C) HaCaT cell (ϕ = 0.2) droplets (PEO 5.5 wt%/DEX 5.5 wt%) and (D to F) HaCaT cell spheroids (PEO 10 wt%/DEX 5.5 wt%) stabilized by 2wt% WP particles. Scale bars are (A, D) 200 µm, (B, E) 100 µm and (C, F) 50 µm.

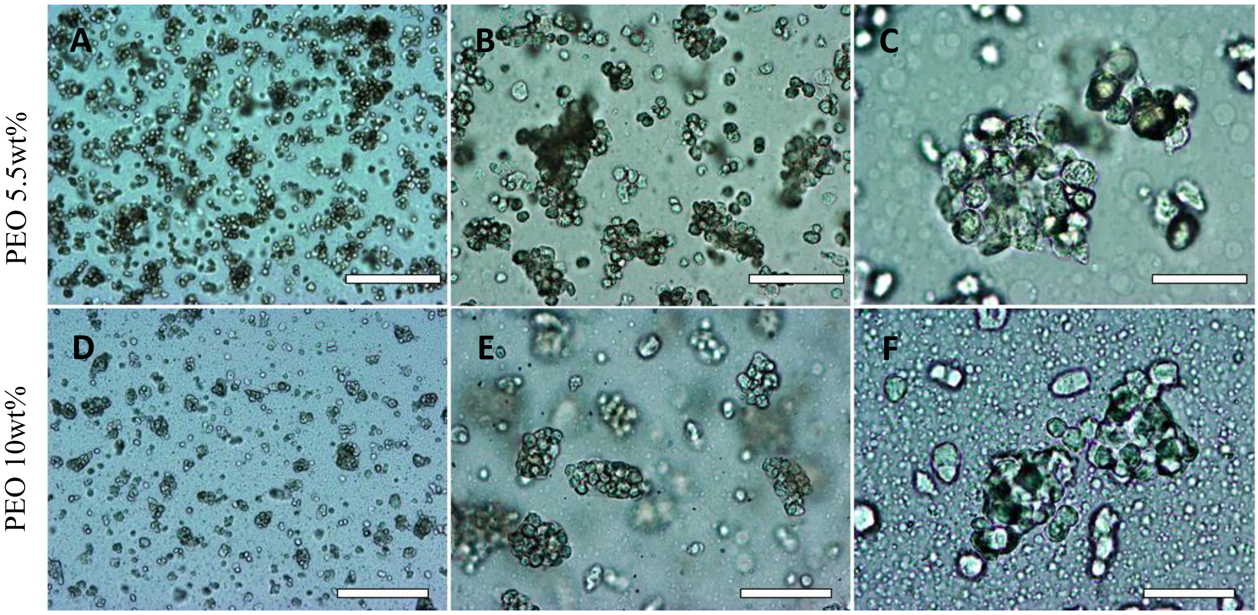

Figure S5. Optical microscopy images of (A to C) HaCaT cell (ϕ = 0.25) droplets (PEO 5.5 wt%/DEX 5.5 wt%) and (D to F) HaCaT cell spheroids (PEO 10wt%/DEX 5.5 wt%) stabilized by 2wt% WP particles. Scale bars are (A, D) 200 µm, (B, E) 100 µm and (C, F) 50 µm.

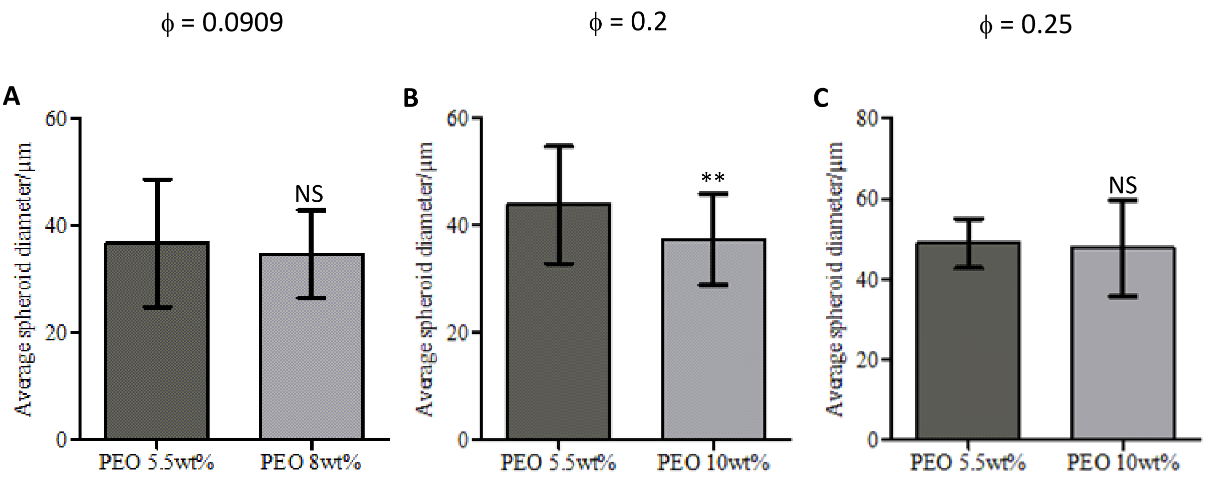

Figure S6. Average HaCaT spheroid diameter for emulsions (A) PEO 5.5wt or PEO 8wt% $\phi_{HaCaT} = 0.0909$, (B) PEO 5.5 wt% or PEO 10 wt% $\phi_{HaCaT} = 0.2$ and (C) PEO 5.5 wt% or PEO 10wt% $\phi_{HaCaT} = 0.25$. Data were obtained by optical microscopy measurements of droplets for each micrograph with ImageJ Software. (Student's t-Test, NS: non-significant, **: $P < 0.01$).

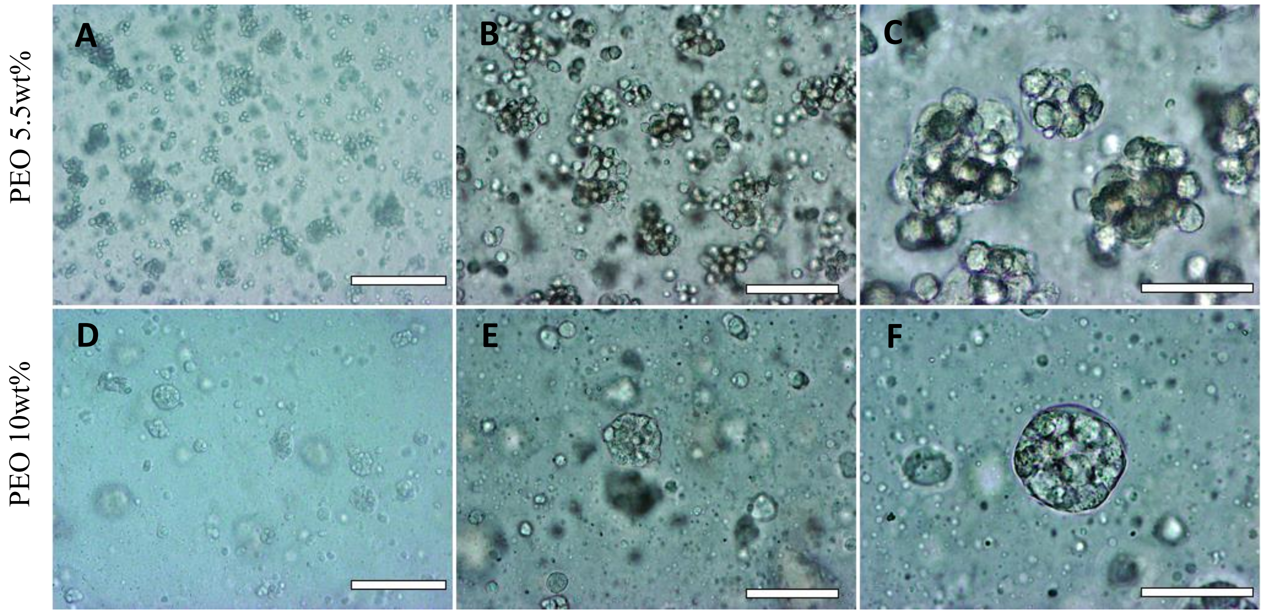

Figure S7. Optical microscopy images of (A to C) HaCaT cell droplets (PEO 5.5 wt%/DEX 5.5 wt%) and (D to F) HaCaT cell spheroids (PEO 10 wt%/DEX 5.5 wt%) stabilized by 2 wt% WP particles. $\phi_{HaCaT} = 0.25$ and $\phi_{Dex} = 0.2$. Scale bars are (A, D) 200 μm, (B, E) 100 μm and (C, F) 50 μm.

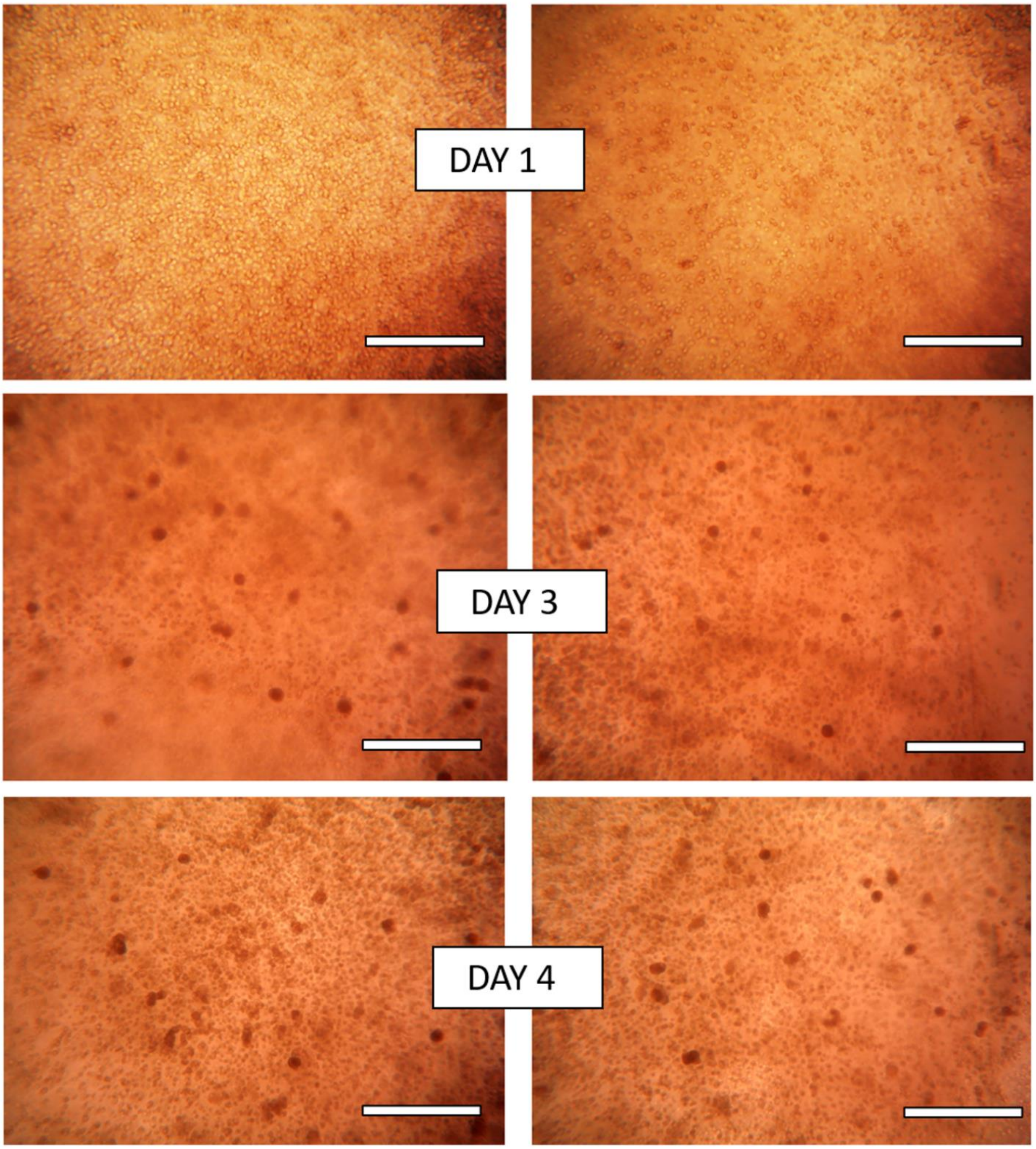

Figure S8. Growth of individual HaCaT cells incorporated with 0.75wt% sodium alginate in DMEM media followed by cross-linking with 1 M $CaCl_2$. The HaCaT cells were cultured in the alginate film for 4 days under DMEM media and images were taken from each well. Scale bars are 200 µm. Note that a very small fraction of the individual HaCaT cells develop into spheroids and growth over the course of 4 days.

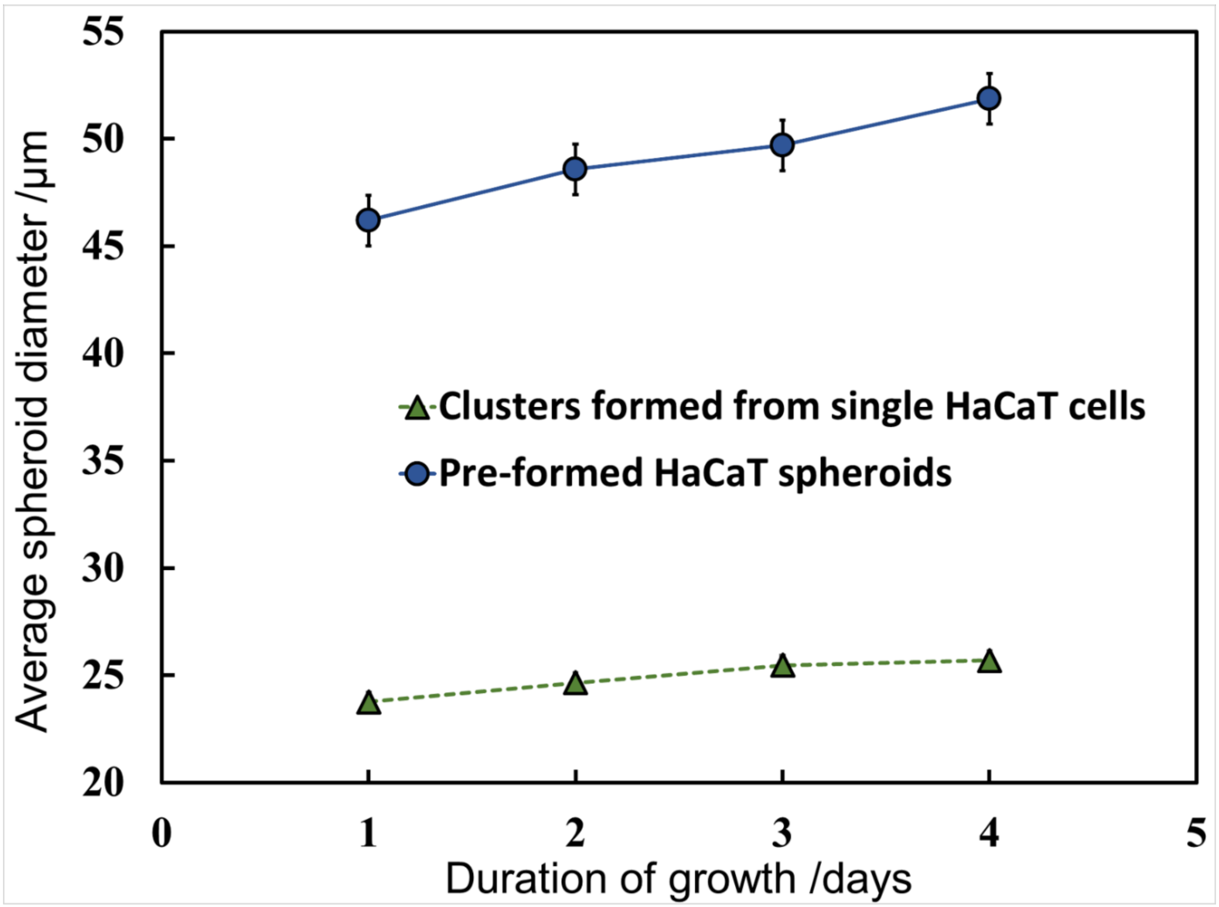

Figure S9. Growth curves of spheroids spontaneously formed from individual HaCaT cells (triangle symbols) and pre-formed HaCaT spheroids by our method (circle symbols) both incorporated within a film of 0.75wt% sodium alginate in DMEM media followed by cross-linking with 1 M $CaCl_2$. The HaCaT cells were cultured in the alginate film for 4 days under DMEM media and images were taken from each well. Note that a lower rate of growth of the individual HaCaT cells that develop into spheroids over the course of 4 days – see typical images of the alginate films with the individual cells growth in Figure S7 (The error bars are within the symbol size).
